# De novo sequence assembly and characterisation of a partial transcriptome for an evolutionarily distinct reptile, the tuatara (*Sphenodon punctatus*)

**DOI:** 10.1186/1471-2164-13-439

**Published:** 2012-08-31

**Authors:** Hilary C Miller, Patrick J Biggs, Claudia Voelckel, Nicola J Nelson

**Affiliations:** 1 Allan Wilson Centre for Molecular Ecology and Evolution, School of Biological Sciences, Victoria University of Wellington, PO Box 600, Wellington, 6140, New Zealand; 2 Infectious Disease Research Centre (IDReC), Institute of Veterinary, Animal & Biomedical Sciences, Massey University, Private Bag 11-222, Palmerston North, New Zealand; 3 Allan Wilson Centre for Molecular Ecology and Evolution, Institute of Molecular BioSciences, Massey University, Private Bag 11 222, Palmerston North, New Zealand

**Keywords:** Illumina sequencing, Transcriptome, De novo assembly, Sex determination, Repetitive elements, Tuatara, Sphenodon punctatus, Reptile genomics

## Abstract

**Background:**

The tuatara (*Sphenodon punctatus*) is a species of extraordinary zoological interest, being the only surviving member of an entire order of reptiles which diverged early in amniote evolution. In addition to their unique phylogenetic placement, many aspects of tuatara biology, including temperature-dependent sex determination, cold adaptation and extreme longevity have the potential to inform studies of genome evolution and development. Despite increasing interest in the tuatara genome, genomic resources for the species are still very limited. We aimed to address this by assembling a transcriptome for tuatara from an early-stage embryo, which will provide a resource for genome annotation, molecular marker development and studies of development and adaptation in tuatara.

**Results:**

We obtained 30 million paired-end 50 bp reads from an Illumina Genome Analyzer and assembled them with Velvet and Oases using a range of kmers. After removing redundancy and filtering out low quality transcripts, our transcriptome dataset contained 32911 transcripts, with an N50 of 675 and a mean length of 451 bp. Almost 50% (15965) of these transcripts could be annotated by comparison with the NCBI non-redundant (NR) protein database or the chicken, green anole and zebrafish UniGene sets. A scan of candidate genes and repetitive elements revealed genes involved in immune function, sex differentiation and temperature-sensitivity, as well as over 200 microsatellite markers.

**Conclusions:**

This dataset represents a major increase in genomic resources for the tuatara, increasing the number of annotated gene sequences from just 60 to almost 16,000. This will facilitate future research in sex determination, genome evolution, local adaptation and population genetics of tuatara, as well as inform studies on amniote evolution.

## Background

The advent of next-generation sequencing technology has made it possible to gather large amounts of genomic information from non-model organisms at a fraction of the time and effort previously required [1]. Transcriptome datasets (i.e. sequences from expressed genes only) can provide a tractable entry into genomic analysis for species which do not have a close relative with a full genome sequence [2]. These datasets comprise a smaller subset of sequence, with fewer repetitive elements, intron or intragenic sequences that make assembly of full genomes difficult. Transcriptomes thus require fewer computational resources for assembly than full genomes, can be annotated by comparison with protein sequences from even distantly related species, and have a high functional information content.

Creation of a reference transcriptome can facilitate whole genome annotation, molecular marker development and studies of development or adaptation in non-model organisms. Previous studies have used transcriptome datasets to identify candidate genes for biologically important traits [3,4], genes involved in adaptation to specific environments [5,6], and to develop microsatellite and single nucleotide polymorphism (SNP) markers for genomic mapping and population genetic surveys [7,8].

The tuatara (*Sphenodon punctatus*) is a species of extraordinary evolutionary and physiological interest, yet has few existing genomic resources. They are the only surviving member of the reptilian order Rhynchocephalia (also known as Sphenodontia), which diverged from other reptilian orders approximately 250 million years ago. Rynchocephalids are regarded as the sister group of the squamates, based on morphological and genetic analyses [9,10] and were globally widespread until the late Cretaceous (65–80 million years ago) [11].

In addition to being one of the most phylogenetically divergent vertebrate lineages, tuatara have several distinctive physiological traits which make them a useful model for studies of genome evolution and development. They have a rare pattern of temperature-dependent sex determination, where males are produced at high temperatures [12]; an unusual thermal biology, being cold-adapted with a low optimal body temperature range for a reptile (16-21°C) [13-15]; and a low metabolic rate [14]. They are also extremely long-lived, probably living to over 100 years [16,17], have a long generation time (sexual maturity at 14 years, mean generation interval 50 years) [18,19], and a low reproductive output, with females reproducing only every four years on average [18].

Tuatara are now restricted to offshore islands around New Zealand, and many populations are small, genetically distinct, and at risk of extinction [20,21]. Climate change poses a particular risk to tuatara populations because of their cold-adapted metabolism and the likelihood of male-biased populations at higher temperatures [22]. Establishing new populations is therefore a central part of tuatara conservation management [20], but requires detailed information on the genetic relatedness of existing populations and adaptation to local environments. Understanding local adaptation, including differences in disease resistance, thermal tolerance, and the interaction of temperature-dependent sex determination and nesting behaviour will be crucial to successful management of tuatara in the face of climate change.

Interest in tuatara genomics is increasing and the construction of a tuatara BAC library has facilitated initial characterisation and mapping of the tuatara genome [23-25]. A limited number of microsatellite, major histocompatibility complex and mitochondrial markers are available for tuatara [26-29], but the species is still poorly characterised at the molecular level. Prior to this study, only 60 partial or full mRNA sequences for tuatara were available on Genbank.

Here we have used Illumina sequencing to assemble a partial transcriptome dataset from a tuatara embryo. Although most transcriptome studies from non-model organisms (e.g. [1,8,30,31]) have used 454 pyrosequencing because of the longer read length (>200 bp) produced from this type of sequencer, several recent studies have successfully performed *de novo* assembly of transcriptome data from Illumina reads ranging from 35 to 100 bp in length [3,32-35]. Although the read lengths produced are shorter, Illumina sequencers produce more sequence, at lower cost, than the 454 platform [36]. The greater sequence coverage obtained with Illumina facilitates the assembly of transcripts and enables rare transcripts to be identified. We assembled 30 million paired 50 bp reads into 32911 contigs, almost half of which could be annotated by comparison with sequences in the NCBI NR or UniGene databases. Our primary goal was to produce a reference set of mRNA sequences for tuatara, which will facilitate annotation of the tuatara genome and future studies of sex determination, local adaptation, genome evolution and population genetics of tuatara.

## Results and discussion

Sequencing of tuatara embryo mRNA on the Illumina Genome Analyzer II yielded a total of 1.5 Gbp of mRNA sequence from approximately 30 million paired-end 50 bp reads. Raw reads are available at the NCBI Short Read Archive under accession number SRA051647.

The Q20 percentage (percent of reads with mean error rate <1%), N percentage (percentage of ambiguous “N” bases), and GC percentage for the raw reads was 90.5%, 0.1% and 52.2%, respectively. Reads were quality filtered by trimming off bases with a Phred quality score <13, and then removing reads that were less than 25 bp long. This resulted in a total of 6,231,241 reads (20%) being removed, leaving 20,036,470 paired reads and 4,050,839 single reads. Mean read length after trimming was 38 bp (median read length 46 bp), and the Q20% increased to 99.9%. Although this quality filtering removed a large amount of sequence, removal of low quality bases is likely to result in a more accurate assembly [37].

### De novo assembly

The trimmed sequence reads were assembled into 35,680 transcripts using Velvet and Oases by merging individual assemblies produced with a range of kmers (21–41). Oases clusters transcript isoforms into putative loci [38]. However, because we aimed to produce a non-redundant set of mRNA sequences for tuatara rather than study alternative splicing, we used a python script to choose the longest and/or highest coverage transcript for each locus prior to merging individual kmer assemblies. Duplicates were then removed with CD-HIT-EST [39]. Thus, when we refer to transcripts in this study, we are referring to the representative transcripts chosen for each locus. Table 1 gives summary statistics for each kmer assembly before and after representative transcripts were chosen, and for the final merged set of transcripts.

**Table 1:** Summary statistics for individual and merged assemblies

**Kmer**	**Assembly**	**No. transcripts >100 bp**	**N50**	**Mean length**	**Max length**	**Total no. bases**
21	Initial	33024	844	525	5689	17,354,832
	Representative	29082	786	501	5659	14,561,997
25	Initial	28723	746	491	5689	14,105,603
	Representative	26715	706	474	5659	12,660,658
29	Initial	26236	615	431	5689	11,307,053
	Representative	25016	590	419	5659	10,488,297
33	Initial	23648	488	363	5584	8,591,562
	Representative	22972	469	355	5584	8,148,996
37	Initial	19180	369	311	5111	5,898,486
	Representative	18821	357	301	5111	5,664,511
41	Initial	12230	281	263	5750	3,218,609
	Representative	12090	273	258	5750	3,122,927
Merged		35680	747	479	5750	17,086,468
**Final**		**32911**	**675**	**451**	**5659**	**14,828,283**
Annotated		15965	927	586	5659	9,357,209

For each kmer, data from both the initial Velvet/Oases assembly (Initial), and the assembly containing only one representative transcript from each locus (Representative) are shown. The “Merged” assembly is the result of merging representative assemblies from different kmers using CD-HIT-EST, the “Final” assembly is after potentially misassembled transcripts were removed, and the “Annotated” set only contains transcripts with a significant BLAST match. Kmer = required length of overlap match between two reads in Velvet; N50 = length-weighted median contig length.

Assemblies at higher kmers (e.g. 29–41) used fewer reads and had lower mean contig length than assemblies at lower kmers (21 and 25), as after quality trimming many reads were shorter than the kmer length for higher kmers, so fewer reads were used in these assemblies. However, assemblies using higher kmers contained transcripts not captured at lower kmers, so the final merged assembly had a greater diversity of transcripts overall. The lower N50 and mean contig length of the merged assembly compared with the 21 and 25 kmer assemblies is due to the addition of shorter sequences from the higher kmer assemblies.

### Assembly validation and annotation

**Figure 1:**
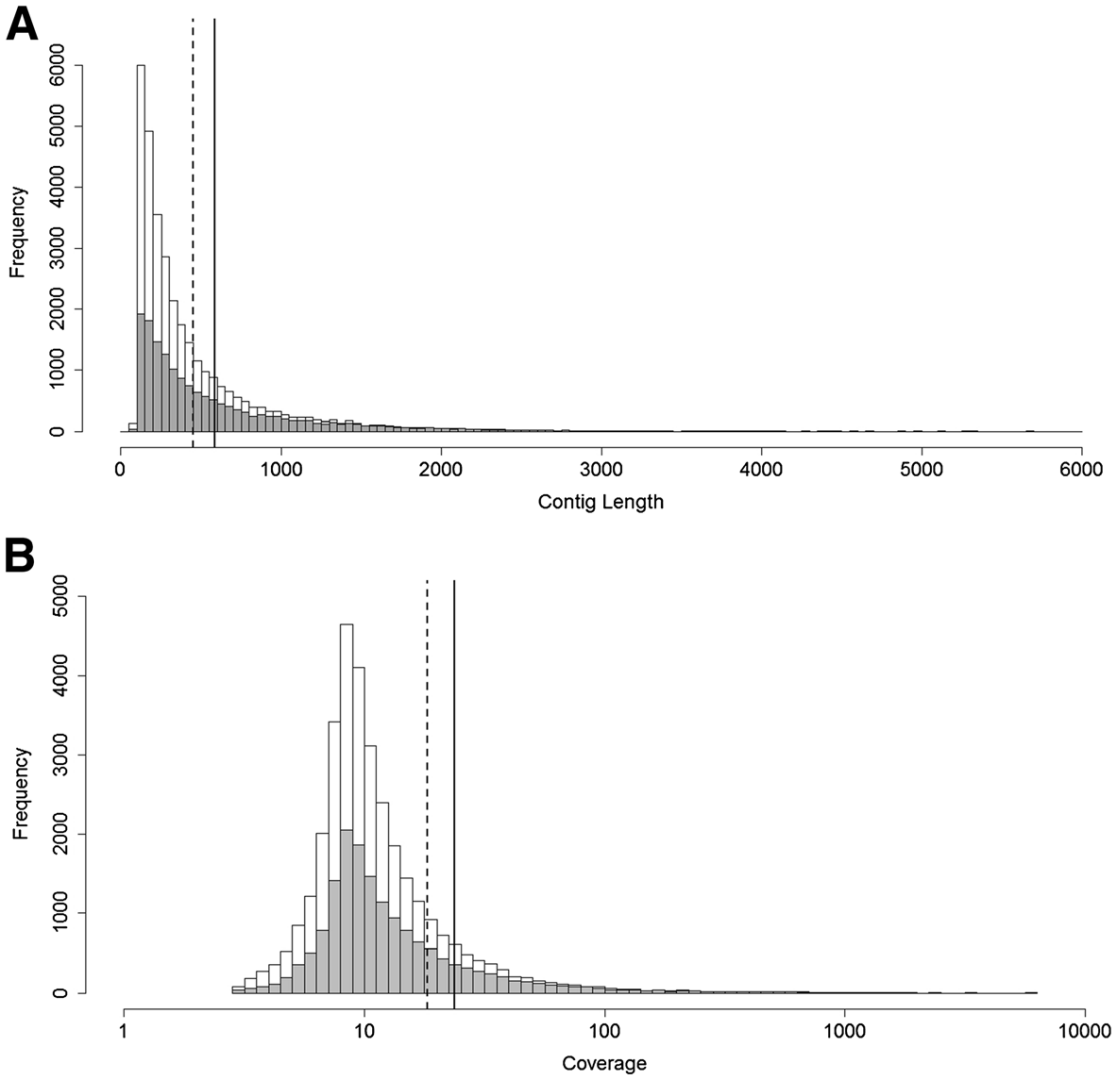
**Length distribution (A) and mean coverage per base (B) of assembled transcripts.** White bars show data from all transcripts (32,911 sequences), while grey bars show data from annotated sequences only (15,965 sequences). The line denotes mean length (**A**) or coverage (**B**) for all transcripts (dotted line), and annotated sequences only (solid line).

Assessing whether transcripts have actually assembled correctly is problematic in the absence of a reference genome, so we employed an internal validation method of mapping quality-trimmed reads back to the assembly in order to identify poor quality and potentially misassembled transcripts. A total of 2,504 transcripts that either had a mean coverage per base of less than 3, or contained coverage gaps, were removed from the assembly. We also removed 265 transcripts that were identified by RepeatMasker as containing ribosomal RNA sequences. The final dataset, which was used for all subsequent analyses, now contained 32,911 transcripts, with an N50 of 675 bp and a mean transcript length of 451 bp (Table 1, “Final”). A FASTA file of these transcripts is available as Additional file 1. Transcripts range from 100 bp to 5,659 bp in length, with 3,264 transcripts over 1 kb (Figure 1A), and a mean coverage per base, from mapping with BWA, of 18.26 (Figure 1B). The mean transcript length in our study was similar to that obtained in transcriptome studies for non-model organisms using 454 data (e.g. guppy *Poecilia reticulata,* 464 bp [8]; European eel *Anguilla Anguilla,* 531 bp [30]; Flesh fly *Sacrophaga crassipalpis,* 332 bp [40]).

We further validated our assembly by assessing the sequence completeness and information content of our set of transcripts. The Full-Lengther software identified 28.6% of our transcripts as full length (8,818 transcripts), or putative full length (607 transcripts). These transcripts were predicted to contain the entire protein coding region but may have incomplete 5` or 3` untranslated regions. This result indicates that many of our transcripts represent only fragments of genes, and further sequencing will be required to increase the number of full-length transcripts.

BLAST searches were performed to assess the percentage of transcripts that match known genes in other species, and thus are likely to contain valid genes. We used megablast to query the 60 known tuatara mRNA sequences, and tblastx to compare our transcripts with the green anole lizard, chicken and zebrafinch UniGene sets. Thirty-four transcripts had significant matches to a total of 32 different tuatara mRNA sequences. Most of the existing tuatara mRNA sequences are only partial coding sequences, and in many cases our sequences only overlapped with a portion of this previously isolated sequence. However in the regions of overlap, 73% of transcripts had 95% or greater sequence identity. It is difficult to assess whether sequence differences between our transcripts and existing tuatara sequences represent sequence errors. In many cases the differences likely reflect real genetic variation among individual tuatara, or matches with related, but non-homologous genes.

In total, 14,748 transcripts had a significant match to green anole, zebrafinch or chicken UniGene sequences, with 5,183 transcripts matching all three. The highest number of matches was to the chicken UniGene set, with 41.2% (13,556) of our transcripts matching. The zebrafinch UniGene set had the next highest number of matches, with 29.8% (9,822) of our transcripts matching, but only 21.4% (7,029) had matches in the green anole UniGene set. Of the three UniGene sets we used for comparison, the chicken set is most likely to represent a complete transcriptome. This UniGene set is constructed from over 200 individual cDNA libraries and has undergone more than 40 builds, compared with the green anole UniGene set which is constructed from only 7 libraries and has undergone only 3 builds. Thus, the low number of matches with the green anole UniGene set compared with that of chicken is probably due to the fact that the green anole UniGene set is less complete than that of chicken.

Of the 13,556 tuatara transcripts which matched the chicken UniGene set, 6,968 matched unique chicken genes suggesting that our transcripts represent nearly 7,000 individual genes. The assembly method we used where assemblies produced from multiple k-mers are merged, may result in the proliferation of duplicate contigs. Although using CD-HIT-EST should have removed the majority of these duplicates, there is still a possibility that our dataset contains some redundancy. The BLAST results against the chicken UniGene dataset indicates that we do have instances where multiple transcripts match a single chicken gene, but in most cases these represent transcripts mapping to different parts of the same gene, or paralogous tuatara sequences having the same chicken gene as the best match. Few appear to be the result of duplicates introduced by the assembly process. As the entire chicken UniGene set (build #43) contains 31,576 sequences our dataset covers about 22% (= 6,968/31,576) of the transcriptome. This should be regarded as a minimum estimate, as the large evolutionary distance between tuatara and chicken (~285 MYA, [10]) means that many tuatara orthologues of chicken genes would be too diverged to align.

We further annotated our sequences by searching the NCBI non-redundant (NR) protein database using blastx. We found that 12,930 sequences (39.3%) had matches with an e-value of less than 1e^-5^. The species with the highest number of top BLAST hits was the green anole lizard (2,726 sequences)*,* followed by chicken (1,661 sequences), zebrafinch (1,143 sequences) and turkey (1,087 sequences).

We compiled a dataset of “annotated” sequences from those that had a significant match (e value less than 1e^-5^) to either the NCBI non-redundant (NR) protein database, the chicken, zebrafinch or green anole UniGene sets, or to previously known tuatara transcripts. This dataset contained 15,965 sequences, with an N50 of 927, mean length of 586 bp, and mean coverage per base of 23.73 (Figure 1, solid bars). Of these sequences, 4,421 were full-length (27.7%). The longer mean length and N50, and higher mean coverage of this dataset shows that sequences with a BLAST match were longer and likely to be of higher quality than those which did not match any known sequences. The un-annotated sequences may still be valid genes, but may have undergone a large amount of sequence divergence or be short fragments of genes. Additional file 2 shows the top BLAST hits to each database for each transcript.

The percentage of sequences that match known genes in our study (48.5% matching either a UniGene set or NCBI NR, and 39.3% matching NR only) compares favourably with other studies on non-model organisms, despite the fact that the tuatara is the most evolutionarily distinct vertebrate for which a transcriptome has been sequenced, sharing a most recent common ancestor with its closest relatives (lizards and snakes) about 250 million years ago. Other *de novo* transcriptome assembly studies on non-model organisms have reported BLAST matches for 20-46% of sequences, with e value thresholds ranging from 1e^-3^ to 1e^-6^[8,30,31,41-45]. Among other reptile transcriptome studies, 35.7% of Burmese python, and 34% of garter snake transcripts could be annotated using blastx to chicken, green anole lizard and NCBI-NR databases [43]; and a study of brain transcriptomes from the Nile crocodile, corn snake, bearded dragon and red-eared turtle was able to match 36-46% of transcripts from these species to known sequences in Ensembl and UniGene databases [45].

**Figure 2:**
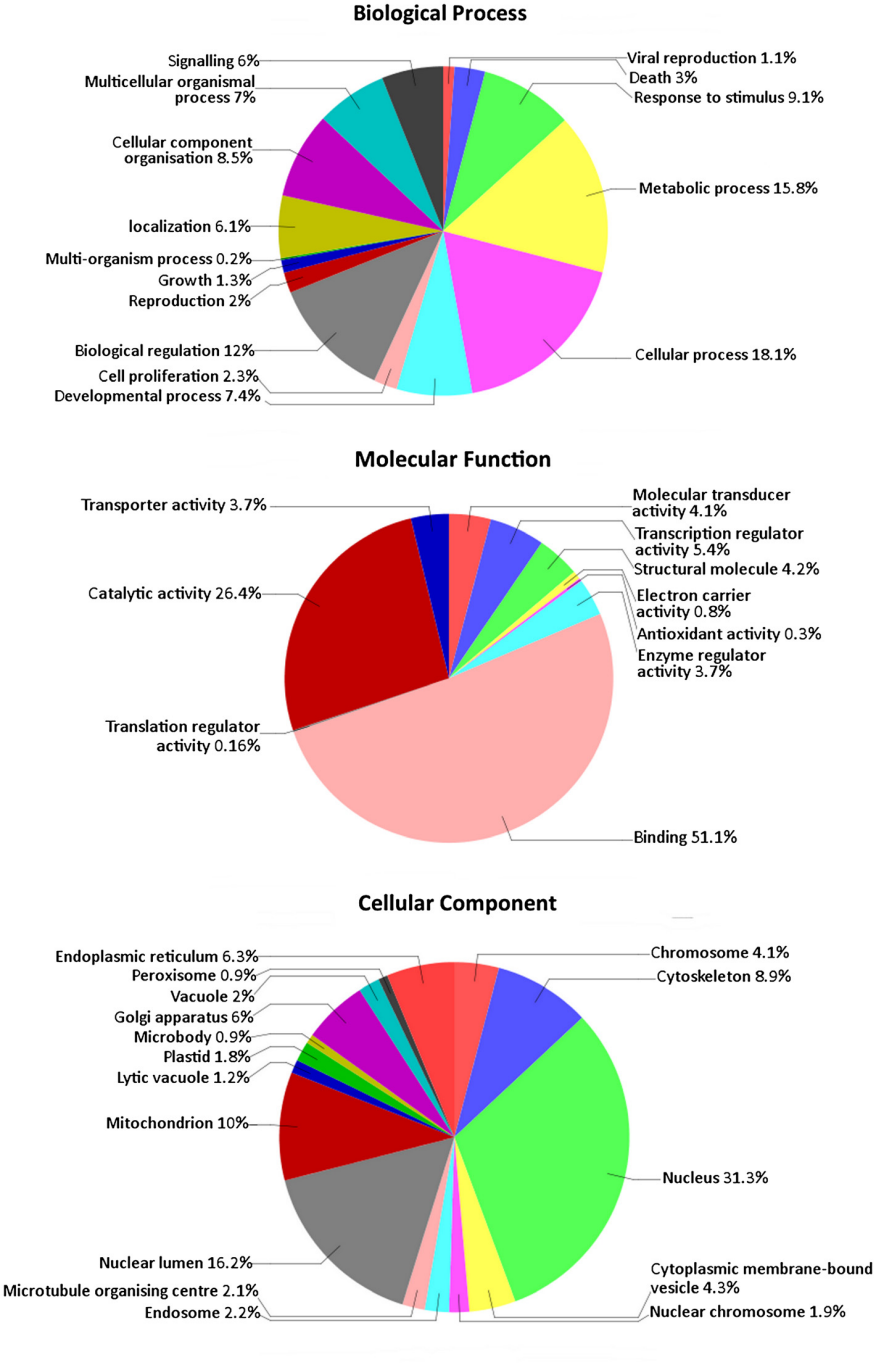
**Gene Ontology (GOslim) assignments for tuatara transcripts.** Level 2 annotations are shown for the biological process and molecular function graphs, and level 7 annotations for the cellular component graph.

We were able to annotate 11,599 of our transcripts with Gene Ontogeny (GO) IDs using Blast2GO. Of these, 9,758 were annotated with a biological process, 10,471 with a molecular function, and 10,458 with a cellular component. A breakdown of the GO annotations within each category is shown in Figure 2. As would be expected in a developing embryo, a large number of transcripts with molecular functions of binding (51.1%) and transcriptional regulation (5.4%) were identified. Our dataset also includes many sequences with biological process annotations that may be relevant to studies of sex determination and adaptation in tuatara, including metabolism (15.8%), development (7.4%), reproduction (2%) and growth (1.3%).

### Candidate genes and repeat content

A keyword search of our BLAST results to the NCBI NR and UniGene databases showed that our dataset contained several candidate genes which may be useful for studies of tuatara local adaptation and sex determination (Table 2). We identified partial sequences for several genes associated with immune function, including major histocompatibility complex (*MHC*) genes and a Toll-like receptor gene.

**Table 2:** Candidate genes for immune function, sex differentiation and temperature-response found in our dataset

**Gene**	**Transcript ID**	**Transcript length**	**Match length**	**Match identity**	**Match accession**
**Immune genes**					
MHC class I	29_Locus_17662	136	136	96%	DQ145788
	29_Locus_8295	526	444	91%	ABA42599
	21_Locus_8663	1455	717	90%	ABA42599
	25_Locus_8701	642	567	88%	ABA42600
	33_Locus_20320	141	138	86%	ABA42600
	25_Locus_21578	159	105	62%	ABA42599
	21_Locus_10540	222	216	50%	ABB92561
MHC class II β chain	21_Locus_1538	921	420	99%	DQ124231
	29_Locus_1474	231	231	94%	DQ124232
MHC class II α chain	21_Locus_7902	232	222	74%	AF256650
	29_Locus_2120	133	111	92%	AF256650
MHC class II DM α chain	21_Locus_1203	963	648	43%	AEC52935
	21_Locus_6166	155	144	60%	ACY01474
Toll-like receptor 2	21_Locus_29589	125	123	98%	ABU95017
**Putative sex-differentiation genes**					
Sox8	25_Locus_11477	766	222	97%	AAO39011
	25_Locus_1895	162	162	93%	ABB02374
	21_Locus_14808	309	252	79%	AAO49746
Sox 9	21_Locus_21814	173	173	100%	AY168558
	21_Locus_18139	167	165	98%	ACU12331
	21_Locus_24105	142	67	100%	AY168558
Dax1	21_Locus_20366	134	117	92%	ABQ88373
**Temperature-responsive genes**					
CIRBP	37_Locus_775	500	252	82%	XP_003224509
HSP27	21_Locus_6322	1028	543	82%	XP_002190077
	33_Locus_379	827	606	75%	XP_002194703
HSP40 (DnaJ)	21_Locus_336	1887	1233	97%	NP_001005841
	21_Locus_2214	2001	1014	83%	XP_003217107
HSP47	25_Locus_2437	1689	1212	90%	BAF94140
HSP70	21_Locus_6649	1124	1113	96.5%	AEO13403
	25_Locus_13016	548	429	92%	EAY98319
	21_Locus_13133	369	369	90%	ADD69959
	21_Locus_4641	3563	2406	90%	XP_003210546
	25_Locus_21367	271	270	89%	XP_002193237
HSP70BP	25_Locus_4659	1484	693	81%	NP_001025928
	25_Locus_5331	1431	696	84%	XP_003226240
HSP75	21_Locus_7217	2174	1566	90%	BAF94145
HSP90	33_Locus_89	2694	1230 + 372	98%	AF275719
	21_Locus_16233	979	975	90%	BAD95027
	21_Locus_31051	139	138	96%	AAD11550

The accession number for the top BLAST hit is given. Not all matches for heat-shock proteins are shown. CIRBP = Cold-inducible RNA binding protein.

A search for genes known to be involved in sex determination and differentiation found *Sox8*, *Sox9,* and *Dax1*, but no sign of *DMRT1*, *WT1*, *Sf1*, *Aromatase*, *Anti-mullerian Hormone*, *WNT4*, or *FoxL2*. The initial trigger for temperature-dependent sex determination in reptiles is not known, and many of the genes listed above are expressed as the gonads are differentiating into male or female forms [46,47]. The embryo we used in this study was sacrificed during the thermosensitive period, the window of time when temperature determines the sex of the embryo, but before full differentiation of the gonads. By switching the embryo from female to male-producing temperatures for one week during this time we had hoped to encourage expression of both male and female differentiating transcripts. However, the lack of major sex differentiating genes in our dataset suggests that the sex-differentiating pathway was not fully activated when our embryo was sacrificed. It is also possible that switching from female to male-producing temperatures switched off the expression of female-differentiating genes. The presence of *Sox* genes and *Dax1* in our dataset indicates that these may play an early role in sex differentiation in tuatara, but we were not able to determine whether these genes are expressed specifically in developing gonadal tissue.

We also searched for genes known to be upregulated in response to temperature, including cold-inducible RNA binding protein (CIRBP) and heat-shock proteins. We found a single transcript matching the full-length CIRBP gene. This transcript had high coverage (mean coverage per base 1,589), suggesting it is highly expressed. CIRBP is known to be induced by low temperature in other species and acts as a translational repressor [48]. In snapping turtles it is differentially expressed in the gonads early in the sex determining period, indicating a potential role in temperature-dependent sex determination [47]. We also found more than 20 transcripts matching seven different types of heat-shock protein (*Hsp27*, *Hsp40*, *Hsp47*, *Hsp70*, *Hsp70bp*, *Hsp75* and *Hsp90*). Heat shock proteins have also been implicated in temperature-dependent sex determination, with sexually dimorphic expression of *Hsp27*, *Hsp70* and *Hsp90* observed in American alligator gonadal tissue [49]. In order to further elucidate the role of these and other potential sex determination triggers, future work should focus on transcriptome analysis of genital ridge cells from both male and female embryos, both before, during and after the thermosensitive period.

The tuatara genome is known to contain a large number and diversity of repetitive elements [50], so we used RepeatMasker to determine whether these are transcribed into RNA. A total of 1,803 repetitive elements and 1,072 regions of low complexity were identified in our dataset, comprising 1.4% of the overall sequence (Table 3). Retroelements made up 0.75% of the sequence (792 elements), including 488 long interspersed repeats (LINEs), 177 short interspersed repeats (SINEs) and 127 long terminal repeat (LTR) elements. The most commonly found retroelement was the chicken repeat 1 (CR1, 342 elements), but 26 L1 and 119 L2 elements were also present. CR1, L1 and L2 have previously been identified in the tuatara genome, as have all of the LTR elements identified in this study [50]. Other repetitive elements found in this study included DNA transposons (7 types), satellites and simple sequence repeats (microsatellites). Repetitive elements account for 30% of the green anole lizard genome [51] and 30-50% of mammalian genomes [52], and are known to modulate the expression of nearby genes, for instance by providing an alternative promoter or by repressing transcription [52,53]. Although many repetitive elements are not expressed, transcriptionally active repetitive elements have been identified in other transcriptomes, including those of plants [41], *Drosophila*[54], humans [55] and snake venom glands [4] and liver [56]. Reptile genomes contain a wider variety of repetitive elements than those of birds or mammals, and tuatara appear to have a higher diversity of repeat types than other birds and reptiles studied to date, which is consistent with their low metabolic rate, large genome size and long divergence time from other reptiles [50]. The number of transcribed repetitive elements in the tuatara genome may be much higher than what we measured here, as repetitive DNA is known to be difficult to assemble, especially from short reads. Thus many of the reads that were not used in our assembly might contain repetitive elements.

**Table 3:** Summary of repeats identified in tuatara transcripts

**Repeat type**	**Number of elements**	**Length occupied (nucleotides)**	**Percentage of sequence**
Retroelements	792	112,220	0.75
SINES:	177	19,814	0.13
LINES:	488	76,335	0.51
L2/CR1/Rex	459	72,305	0.48
RTE/Bov-B	3	160	0.001
L1/CIN4	25	3,655	0.025
LTR elements:	127	16,071	0.11
Bel/Pao	1	54	0.0004
Ty1/Copia	6	1,490	0.01
Gypsy/DIRS1	65	10,075	0.07
Retroviral	52	4,252	0.03
DNA transposons	105	11,133	0.07
Hobo-Activator	30	1,698	0.01
Tc1-IS630-Pogo	6	732	0.005
PiggyBac	15	2,039	0.01
Tourist/Harbinger	1	59	0.0004
Unclassified interspersed repeat	15	1,187	0.008
Small RNA*	61	7,028	0.05
Satellites	44	5,917	0.04
Simple repeats	786	30,947	0.21
Low complexity	1072	48,337	0.32

* tRNA and snRNA sequences (rRNAs removed).

Using Msatcommander, we found 137 dinucleotide, 55 trinucleotide and 23 tetranucleotide microsatellites longer than 20 bp, across 208 transcripts. Eight transcripts contained more than one microsatellite. This shows that our dataset is a rich source of microsatellites which could potentially be useful for studying population genetic structure, demography and the genetic basis of local adaptation in tuatara. Previous studies have characterised a total of 18 microsatellite markers for tuatara [26,57,58] but their location in the genome, and potential linkage with functional genes is unknown. Although the microsatellites identified here will require further testing to determine their utility as markers, our dataset has the potential to increase the number of markers available for tuatara by several-fold, and to provide a source of markers linked to known genes which may be useful for assaying functional variation and detecting selection [2].

## Conclusions

Our study provides a first insight into the tuatara transcriptome, increasing the number of annotated tuatara mRNA sequences from 60 to almost 16,000. Our results show the utility of using Illumina short-read data to assemble a transcriptome for a non-model, evolutionarily distinct organism. Even though the read length in our study was short (25–50 bp after removal of poor quality data), the depth of coverage was sufficient to assemble more than 9,425 full-length transcripts and an additional 23,486 partial transcripts, with a mean transcript length similar to that obtained from transcriptomes produced from 454 data and a large number of long transcripts (3264 greater than 1 kb in length).

Although our assembly appears to be of reasonable quality based on transcript metrics such as N50, mean length, and coverage, determining whether transcripts are in fact correctly assembled poses a particular challenge. Not only are tuatara 250 million years removed from their closest relatives the squamate reptiles [10], but reptiles are poorly represented in genome studies in general. At the time of writing, a full genome sequence is only available for one reptile, the green anole lizard [51], and transcriptome data is only available for a handful of species, the majority of which are squamate reptiles [42,43,45]. We employed quality filtering both before and after assembly to increase the probability that only correctly assembled sequences were included in our final dataset. Although this process removed a lot of sequence from our dataset, we were able to match nearly half of the assembled transcripts with known genes, despite the lack of comparative resources, indicating that our dataset contained a high proportion of correctly assembled genes.

As one of the few transcriptomes available for a non-squamate reptile the dataset assembled here will help fill the void of reptile genomic resources and provide key information for studies of amniote evolution. With other reptile genome projects planned or currently in progress [59-62], including one for tuatara (N. Gemmell, pers comm), a new age of reptile genomics is underway [45,63,64]. As one of the earliest-diverging reptile lineages, and the sole representative of one of the four reptilian orders, genomic data for tuatara will be essential for understanding the structure of the ancestral amniote genome and the evolution of key biological systems, such as those involved in sex differentiation, immunity, cold adaptation and metabolism. The tuatara transcriptome assembled in this study can be used as a reference for future studies of gene expression in tuatara and for annotation of the tuatara genome; and contains candidate genes and genetic markers that will be a useful resource for future studies of tuatara biology and ecology.

## Methods

### Sample preparation and sequencing

We used mRNA from an early-stage embryo to build the transcriptome, as we were particularly interested in finding genes associated with temperature-dependent sex determination. This work was carried out under New Zealand Department of Conservation permit #NM-17163-DOA and Victoria University of Wellington Animal Ethics Committee permit #2009R12. A single *Sphenodon punctatus* egg, laid by a captive tuatara of Stephens Island, Marlborough Sounds origin, was incubated at 20°C (female-producing temperature) for 8 weeks after laying, then switched to 23°C (male-producing temperature) for one week before sacrificing. Eggs were switched between incubation temperatures in order to promote expression of both female and male sex-differentiating transcripts, as a previous study indicated that the thermosensitive period for tuatara occurs around this time [65].

The whole embryo was divided into head, trunk and tail – further dissection of individual organs was not practical given the embryo’s early stage of development. Total RNA was extracted from each tissue section using Trizol (Invitrogen). After phase extraction with Trizol and chloroform, an equal volume of 75% ethanol was added to the aqueous phase, and this mixture was added to a HighPure RNA Isolation column (Roche). Purification of RNA from the column was performed according to the manufacturer’s instructions. Illumina sequencing was performed by the Massey Genome Service (Palmerston North, New Zealand). Head, trunk and tail mRNA libraries were prepared using the mRNA-Seq sample prep kit (Illumina, San Diego, CA, USA), and pooled in equimolar amounts before loading onto one lane of an Illumina flow cell. Paired end sequencing was performed on an Illumina Genome Analyzer II, and sequences were initially processed using the Illumina Pipeline Software.

Sequences were filtered for low quality reads using the DynamicTrim and LengthSort Perl scripts within SolexaQA [37]. These scripts trimmed each read to the longest contiguous read segment for which the quality score at each base was greater than p = 0.05 (approximately equivalent to a Phred score of 13), and then removed sequence reads shorter than 25 bp respectively. Where one sequence of a pair was removed, the remaining sequence was put into a separate file and used as a singleton during *de novo* assembly.

### De novo sequence assembly

Sequence assembly was performed using Velvet v1.1.04 [66] and Oases v0.1.21 [38] using a coverage cutoff of 5 and discarding transcripts shorter than 100 bp. Comparisons of short-read assembly programs have shown these two programs to be effective at producing quality assemblies of short-read transcriptome data [3,34]. Velvet uses de-Bruijn graphs for sequence assembly and is specifically designed for short-read sequences. Oases takes the preliminary assembly produced by Velvet and clusters the contigs into loci, then exploits the read sequence and pairing information to produce transcript isoforms. Thus, one or more transcript isoforms may be present for each locus.

Because transcriptome coverage is highly variable depending on the expression level of the gene, there is no one kmer length that will provide an optimal assembly for a transcriptome [67]. Highly expressed transcripts will assemble better with high kmer lengths, while poorly expressed transcripts will be better assembled if low kmer lengths are used. Thus, we ran Velvet and Oases using a range of kmers [21,25,29,33,37,41], and then merged these assemblies to produce a non-redundant set of contigs. Assemblies were merged using a variation on the “additive multiple-k” method proposed by Surget-Groba and Montoya-Burgos (2010), which uses CD-HIT-EST [39] to cluster transcripts on the basis of sequence similarity and retain only the longest transcript from each cluster. As our aim, in the first instance, was to produce a non-redundant set of mRNA sequences for tuatara rather than study alternative splicing, we chose one representative transcript from each locus prior to clustering. This was done using a python script (Adrian Reich, pers comm.) which chooses the best transcript on the basis of coverage and sequence length. The representative transcripts for each kmer assembly were then combined into one fasta file and clustered using CD-HIT-EST with a sequence similarity cut-off of 95%.

### Assembly validation and annotation

In order to identify potentially misassembled transcripts, the reads were mapped back to the assembled transcripts using BWA version 0.5.9rc1 [68]. Paired and single reads were mapped separately then the resulting BAM files were merged using Samtools version 0.1.17 [69]. Mapping results were visualised using the Integrative Genomics Viewer (IGV) version 2.0 [70]. The genome Coverage Bed application within BED Tools version 2.12.0 [71] was used to calculate read coverage at each base. Transcripts with a mean coverage per base of less than 3 were removed from the assembly, as were transcripts that appeared to have been misassembled (i.e. two contigs have been incorrectly joined in one transcript). Where transcripts have been misassembled there will be a drop in coverage over the incorrectly assembled region, as few reads will span this region. To identify and remove these transcripts from our dataset, we used a custom Perl script which identified transcripts containing bases where the coverage dropped to one tenth of the mean coverage for that transcript.

Sequence completeness was assessed using Full-Lengther v0.15 [72], which uses a combination of BLAST searches against UniProt and the NCBI non-redundant (NR) database, and ORF prediction to assess whether transcripts contain the entire protein coding region.

The information content of our assembly was assessed using BLAST searches against existing tuatara mRNA sequences and UniGene sets for the green anole lizard*,* zebrafinch and chicken. UniGene sets were downloaded from Genbank and BLAST searches were performed using tblastx with an e-value cut-off of 1e^-5^. BLAST searches against tuatara mRNAs were performed using megablast with an e-value cut-off of 1e^-5^.

BLAST searches against the NCBI NR protein database were performed using Blast2GO version 2.5.0, using blastx with an e-value cut-off of 1e^-5^. All BLAST results were saved as XML files and uploaded into a MySQL database to facilitate calculation of summary statistics and searches for specific genes. Candidate genes were identified by searching the NCBI NR and UniGene BLAST results for key words.

Gene ontogeny (GO) IDs were assigned to sequences using Blast2GO, based on the blastx output. Annotation was performed using an e-value cut-off of 1e^-3^, an annotation score cut-off of 45, and a GO weight of 5. GO annotations were grouped into GO-slim terms to simplify the output for producing combined graphs. Combined graphs for biological process, molecular function and cellular component were then generated and nodes containing less than 10 sequences were filtered out.

Transcripts containing repetitive elements were identified using RepeatMasker version open-3.3.0 [73]. Repeatmasker was run in default mode with rmblastn version 2.2.25+, Repbase Update 20110419, and RM database version 20110419. The query species was listed as “other vertebrate”, and a GC content of 46-48% was specified. The transcripts were further screened for di, tri and tetra-nucleotide microsatellite repeats using msatcommander [74]. The number of repeat units was set at 10, 7 and 5 for di, tri and tetra-nucleotide repeats, respectively, so only microsatellites greater than 20 bp were identified.

## Competing interests

The authors declare that they have no competing interests.

## Authors’ contributions

HM and NN designed the study. PB assisted with bioinformatics analyses, CV prepared sample and facilitated Illumina sequencing, NN procured sample and performed embryo incubations. HM performed bioinformatics analyses and drafted the manuscript, and all authors contributed to editing the manuscript. All authors read and approved the final manuscript.

## Supplementary Material

Additional file 1 FASTA file of assembled transcripts (after removal of potentially misassembled transcripts). Click here for file

Additional file 2 **Comma-separated file (.csv) of BLAST results.** The top BLAST hit against NCBI-NR, and *Anolis*, zebrafinch and chicken UniGene sets is shown, as is the result of the Full-Lengther analysis. (CSV 8736 kb) Click here for file
